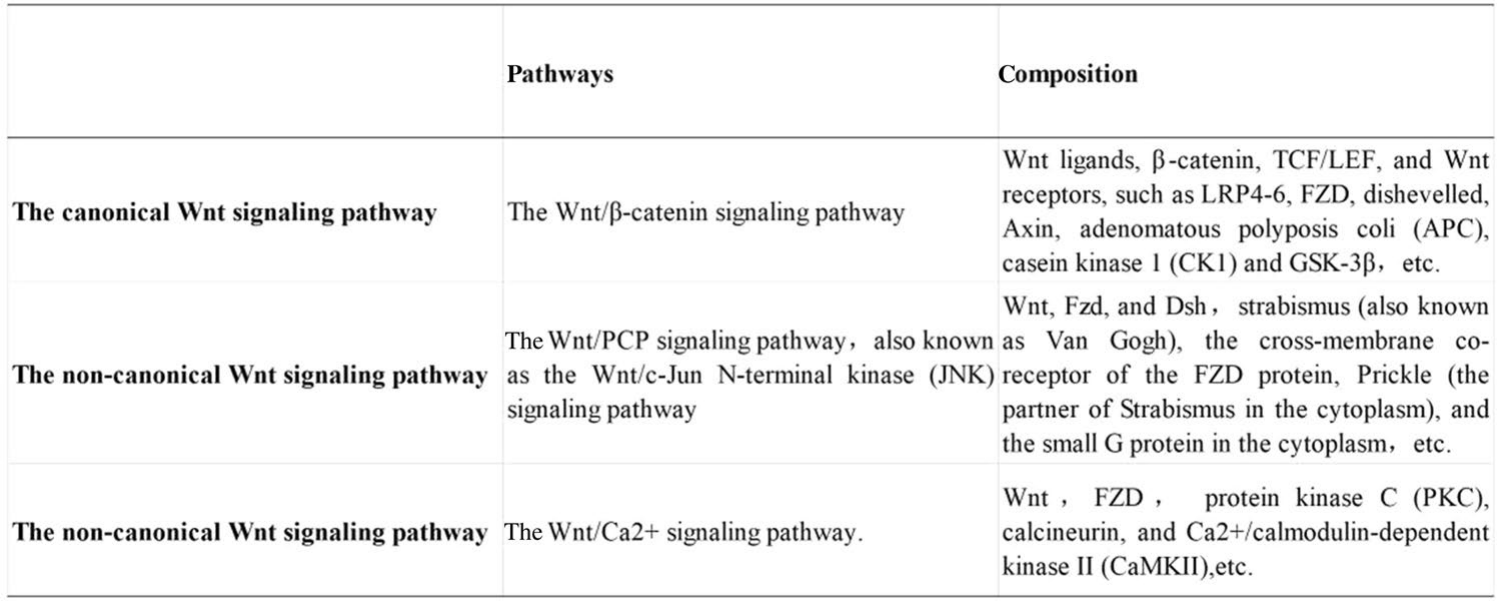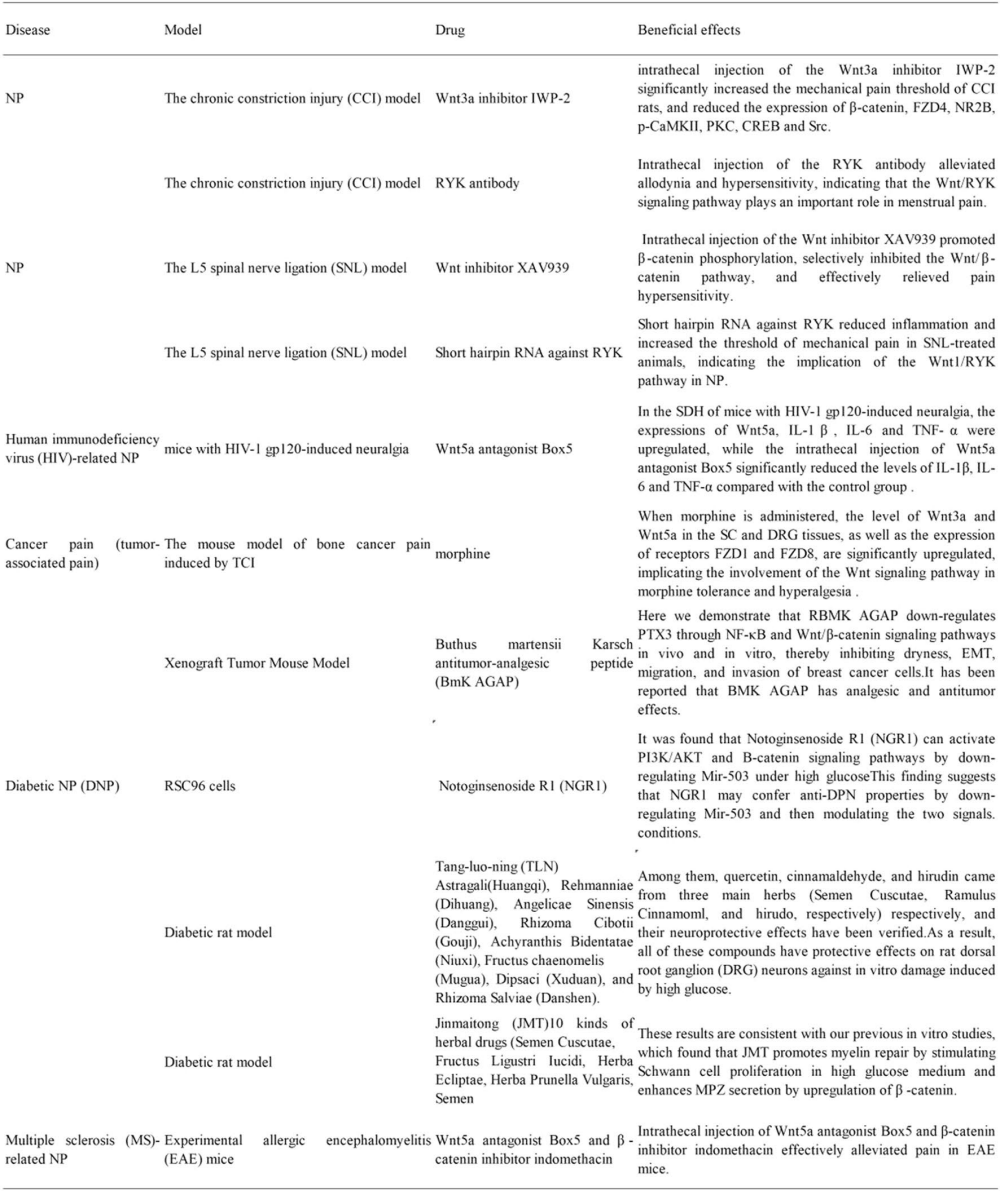# Wnt Signaling Pathways: A Role in Pain Processing

**DOI:** 10.1007/s12017-021-08700-z

**Published:** 2022-01-24

**Authors:** Yiting Tang, Yupeng Chen, Rui Liu, Weidong Li, Baojin Hua, Yanju Bao

**Affiliations:** 1 Guang’anmen Hospital, China Academy of Chinese Medical Sciences, Beixiange 5, Xicheng District, Beijing, 100053 China; 2 Beijing University of Chinese Medicine, Beijing North Third Ring Road No. 11, Chaoyang District, Beijing, 100029 China; 3 Department of Oncology, Guang’anmen Hospital, China Academy of Chinese Medical Sciences, Beixiange 5, Xicheng District, Beijing, 100053 China

**Keywords:** WNT signaling, Pain processing, Neuropathic pain, Cancer pain, Diabetic neuralgia

## Abstract

The wingless-related integration site (Wnt) signaling pathway plays an essential role in embryonic development and nervous system regulation. It is critically involved in multiple types of neuropathic pain (NP), such as HIV-related NP, cancer pain, diabetic neuralgia, multiple sclerosis-related NP, endometriosis pain, and other painful diseases. Wnt signaling is also implicated in the pain induced by sciatic nerve compression injury and selective spinal nerve ligation. Thus, the Wnt signaling pathway may be a potential therapeutic target for NP.

## Introduction

The International Society for Pain Research defines neuropathic pain (NP) as caused by injury or disease of the somatosensory nervous system. The clinical features of NP include spontaneous pain, hyperalgesia, pain hypersensitivity, abnormal sensation, etc. (Fernandes et al., 2018). NP is a common and chronic condition that seriously affects more than one million people worldwide (Zilliox, 2017). Despite decades of research on pain treatment and management, many patients suffering from chronic pain are still unable to benefit from current therapies. Opioids are the main class of prescription drugs for treating chronic pain; however, opioid-associated side effects and tolerance highlight the need for new therapeutic options for pain (Latremoliere & Woolf, 2009). Recent studies have shown that the wingless-related integration site (Wnt) signaling pathway plays a key role in the physiological and pathological processes in the nervous system and is closely related to nerve pain. Therefore, investigations on the Wnt pathway may provide new insights into the pathological mechanism of neuralgia.

The Wnt signaling pathway exists in single-celled organisms (i.e., protozoa) and in mammals. It regulates many physiological processes in the human body, including nervous system development. The Wnt protein family is a group of secreted glycoproteins that function as specific ligands for cells and tissues during brain development and maturation. These proteins participate in neuron formation, cell fate regulation, polarity and migration, axon development, synapse formation, nerve maturation, nerve maintenance, and regeneration (Harrison-Uy & Pleasure, 2012; Salinas, 2012; Willert & Nusse, 2012). In this review, we summarize the regulatory effects of the Wnt signaling pathway in pain pathogenesis, which may contribute to the identification of new interventional targets for pain.

## Introduction of the Wnt Signaling Pathway

### The *Wnt* Gene

The first *Wnt* gene, *Wnt1* (originally named integration site-1), was identified in 1982 as a gene activated by the integration of virus DNA in mouse breast tumors (Nusse & Varmus, 1982). Studies in mouse embryos have demonstrated that the *Wnt* gene plays a crucial role in early development. The name of Wnt is a combination of the *wingless* gene and the *Int1* gene in *Drosophila* (Lerner & Ohlsson, 2015).

The human *Wnt* gene is located on chromosome 12q13 and encodes proteins that are typically 350–400 amino acids in length, including 23–24 conserved half-sarcosine residues and a signal peptide (Daniels et al., 2001). The *Wnt* genes are usually defined by sequence homology using BLAST sequence analysis (Ooyen et al., 1985). So far, over 100 *Wnt* genes have been identified and are sorted into 13 subfamilies: Wnt1–Wnt11, Wnt16, and WntA. According to their functional characteristics, the *Wnt* genes are also divided into two categories: one category is involved in the classic Wnt signaling pathway and includes Wnt1, Wnt3, Wnt3a, Wnt7a, Wnt7b, Wnt8, etc.; the other category is implicated in the non-canonical Wnt signaling pathway, including Wnt4, Wnt5a, Wnt6, Wnt11, etc. (Wong et al., 1994).

The *Wnt* genes are found in both lower and higher animals, with great diversity across species. However, signal transduction pathways related to the classic Wnt signaling have not been reported in plants or fungi.

### Overview of the Wnt Signaling Pathway

The Wnt signaling pathway is a highly conserved pathway with a wide range of biological functions. It plays a key role in tissue homeostasis, organ formation, and nervous system development (Clevers et al., 2014). Since discovering the first member of the Wnt signaling pathway in the early 1980s, a growing number of studies on Wnt signaling have been carried out in different biomedical fields.

Wnt proteins regulate synapse formation, synaptic transmission, and synaptic plasticity in the nervous system (Shi et al., 2012). In the mammalian central nervous system (CNS), certain Wnt ligands, such as Wnt3a (activating the canonical pathway) and Wnt5a (activating the atypical pathway), are mainly expressed in neurons (Gao, 2019; Shi et al., 2013b; Zhu et al., 2017). Other than the canonical Wnt pathway (Wnt/β-catenin pathway), the non-canonical Wnt pathways include the Wnt/planar cell polarity (PCP) pathway and the Wnt/Ca^2+^ pathway. The Wnt signaling pathways contain multiple proteins that activate different intracellular signaling cascades (Langton et al., 2016). The key components in the Wnt signaling pathways are ligands, regulatory proteins, transmembrane receptors, and nuclear transcription factors. When a Wnt protein binds to a transmembrane receptor, such as lipoprotein receptor-related protein (LRP)5/6 or Frizzled (FZD) family protein, the disheveled (DSH/DVL) is activated, leading to the inhibition of glycogen synthase kinase (GSK)-3β and the accumulation of β-catenin in the cytoplasm. Then, the accumulated β-catenin enter the nucleus, bind to the transcription factor T-cell factor/lymphoid enhancer factor (TCF/LEF)-1 and activate the transcription of downstream genes (Daniels et al., 2001).

### Wnt Ligands and Receptors

Wnt proteins are highly conserved glycoproteins, and a total of 19 Wnt ligands have been identified in mammals (Lerner & Ohlsson, 2015). These proteins play a vital role in nervous system development and pain processing by activating cell-surface receptors in typical and atypical Wnt signaling pathways (Inestrosa & Arenas, 2010).

The Wnt receptors include FZD, LRP, receptor tyrosine kinase-like orphan receptor (Ror), protein tyrosine kinases 7 (PTKs7), G-protein coupled receptor (GPCR), etc. (Farin, 2016) In addition, various intracellular mediators and endogenous antagonists, such as Dickkopf (DKK) and secreted frizzled-related protein (SFRP), can inhibit the activation of the Wnt signaling.

The FZD proteins are seven-transmembrane receptors consisting of ten members: FZD1-10. They contain 120 continuous amino acid segments, a signal peptide sequence, a highly variable hydrophilic region, and an N terminus that specifically binds to the Wnt protein (Cavallo et al., 1998). FZD is involved in both β-catenin-dependent and -independent signal transduction. In the β-catenin-dependent Wnt signaling, FZD interacts with LRP5 or LRP6, transducing external signals into intracellular responses. LRP5 and LRP6 are not required in the β-catenin-independent pathway, including the Wnt/PCP and Wnt/Ca^2+^ signaling pathways (Devenport, 2014) (Komiya & Habas, 2008). Other Wnt receptors are also involved in the pathways mentioned above, including Ror2 and receptor-like tyrosine kinase (RYK).

## The Wnt Signaling Pathway

### The Canonical Wnt Signaling Pathway

Beta-catenin is a multifunctional, cytoplasmic protein that plays a central role in the canonical Wnt signaling pathway by acting as a secondary messenger (Rao & Kühl, 2010). Therefore, this pathway is also known as the Wnt/β-catenin signaling pathway (Willert & Nusse, 1998).

**Fig. 1:**
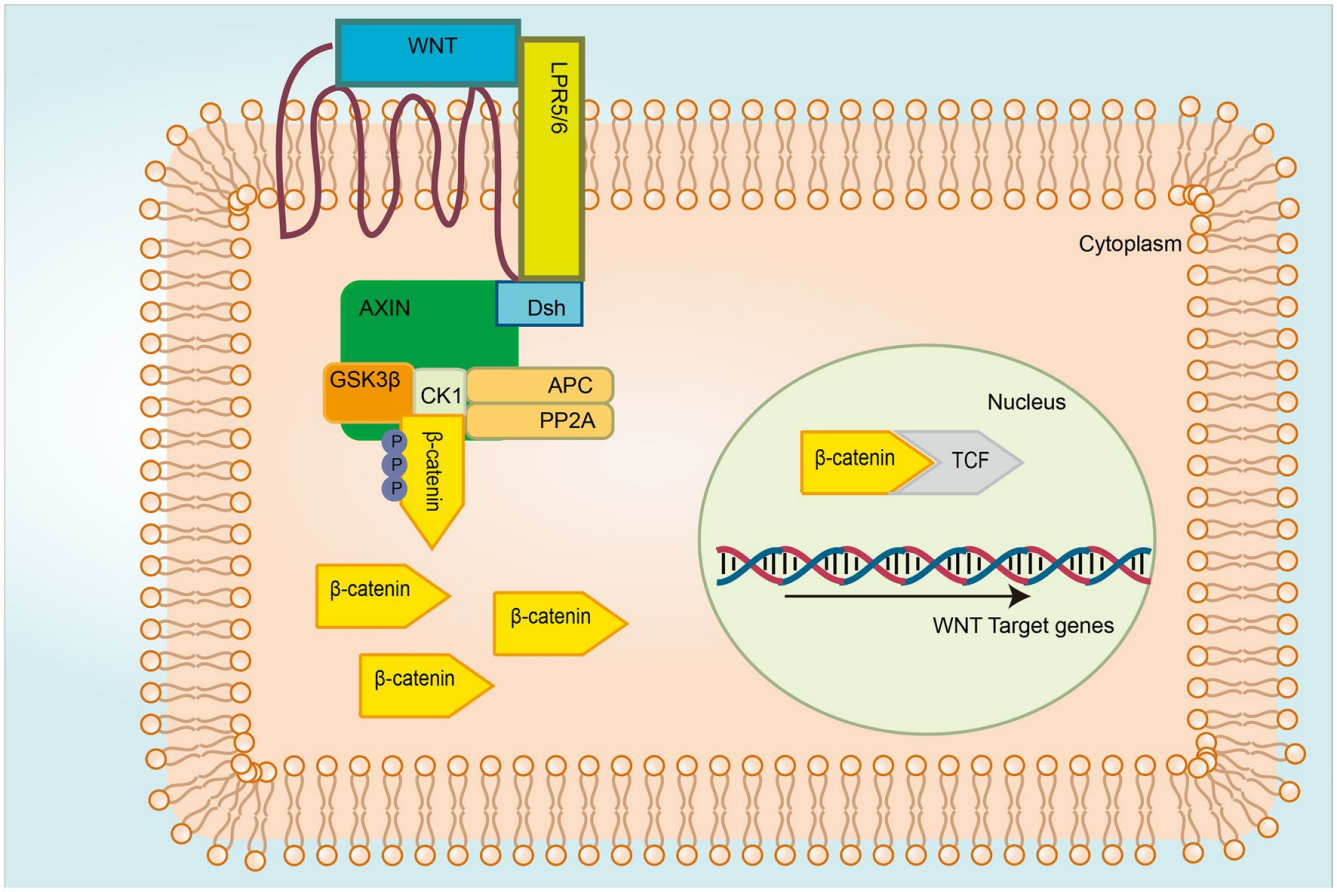
Overview of the canonical Wnt signaling pathways—Wnt ON. The Wnt ligand binds to FRZ and LRP5/6, activates the adaptor protein (DVL), and recruits the Axin complex to the LRP co-receptor. The activated DVL inhibits the destruction of the complex, allowing the accumulation and nuclear translocation of β-catenin, which then forms molecular complex 1/LEF-1 with TCF and initiates the expression of Wnt target genes

The Wnt/β-catenin signaling pathway consists of Wnt ligand, β-catenin, TCF/LEF, Wnt receptor, disheveled, Axin, adenomatous polyposis coli (APC), casein kinase 1 (CK1), and GSK-3β (van Amerongen & Nusse, 2009). The canonical Wnt signaling pathway is activated by binding the Wnt ligand to the FZD receptor and LRP5/6. This interaction activates cytoplasmic effector DSH and therefore prevents the expression and activation of Axin, APC, CK1, and GSK-3β complex (Stamos & Weis, 2013). The inhibition of GSK-3β prevents β-catenin from degradation. The accumulated β-catenin enters the nucleus, replaces the Groucho in the co-repressor/transcription factor T-cell/lymph enhancer factor (Groucho/TCF/LEF) complex, interacts with TCF/LEF, and eventually activates the transcription of Wnt target genes (Lerner & Ohlsson, 2015). The overview of the canonical Wnt signaling pathway is shown in Fig. 1.

**Fig. 2:**
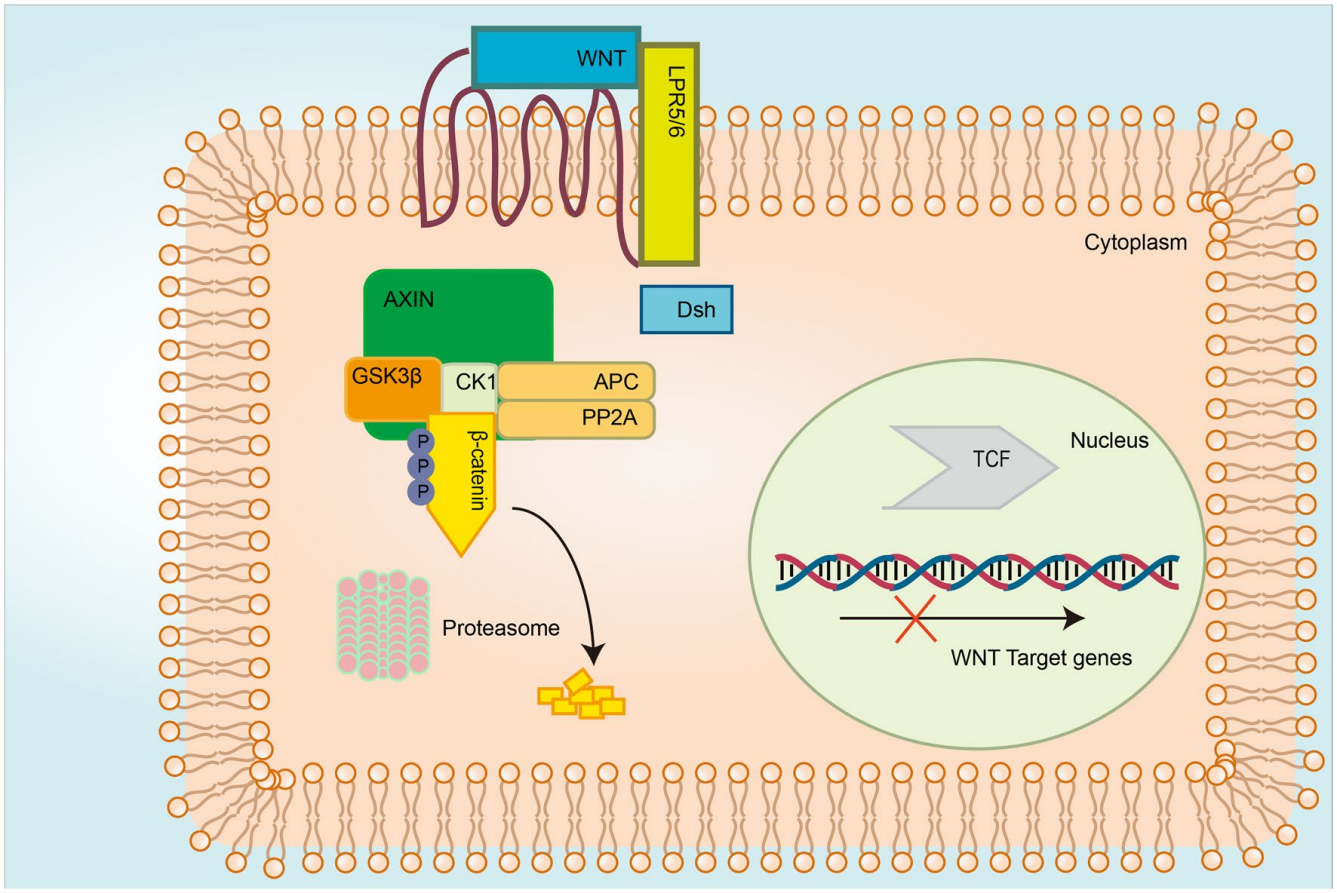
Overview of the canonical Wnt signaling pathways—Wnt OFF. Beta-catenin is mediated by the ubiquitin pathway in the absence of Wnt stimulation. Beta-catenin forms a destruction complex with Axin, APC, GSK-3, CK1 and protein phosphatase 2A. Beta-catenin is phosphorylated by GSK-3 and CK1. After phosphorylation and ubiquitination, β-catenin is degraded by the proteasome. The elimination of cellular β-catenin depletes its pool to translocate to the nucleus, and thus, inhibits the expression of Wnt target genes

Degradable and antagonist proteins regulate the cytoplasmic level of the key factor catenin beta 1 (CTNNB1). The antagonists for the Wnt/β-catenin signaling pathway include CK1, DSH, GSK-3β, and glucan-binding protein (GBP). When no Wnt ligand binds to cell-surface receptors, β-catenin is gradually phosphorylated by CK1 and GSK-3β. The phosphor-β-catenin is ubiquitinated and degraded by Skip-Cullin1-F-box beta-transducin repeat-containing protein (β-TRCP) (Fig. 2) (Azzolin et al., 2014; Stamos & Weis, 2013; Rao & Kühl, 2010). Tankyrase is a regulator of Wnt signaling that phosphorylates Axin by binding to the poly-ADP-ribose chains. Phosphorylated Axin is degraded by the ubiquitin–proteasome system (Guettler & Shapes, 1993). In the presence of Wnt ligands, the cytoplasmic part of FZD binds to DVL, providing a platform for recruiting β-catenin to the degradation complex (Gammons et al., 2016). Once the complex is formed, LRP5/6 is phosphorylated by CK1α and then binds to the complex, inhibiting the degradation of β-catenin (Niehrs & Shen, 2010).

Once the Wnt protein binds to the surface receptor FZD, CK1 is activated and leads to the phosphorylation of DSH, the activation and release of GBP, and the inhibition of GSK-3β activity. In this case, GSK-3β cannot phosphorylate β-catenin, making it unrecognizable to ubiquitin and stably accumulates in the cytoplasm.

### The Non-canonical Wnt Signaling Pathway

The non-canonical Wnt pathways include the Wnt/PCP signaling pathway, also known as the Wnt/c-Jun N-terminal kinase (JNK) signaling pathway, and the Wnt/Ca^2+^ signaling pathway. These pathways are also called the T-cell transcription factor-independent sexual signaling pathway since signal transduction is different from that of β-catenin and transcription factor LEF/TCF (Gómez-Orte et al., 2013; Ho et al., 2012; Roarty et al., 2017).

#### The Wnt/PCP Signaling Pathway

The Wnt/PCP signaling pathway is responsible for establishing cell polarity and regulating directional cell movement. The PCP signaling pathway was originally found in Drosophila, where it controls the planar polarity of the epithelial cells in the eyes, wings, and chest cavity (Adler & Lee, 2001; Mlodzik, 1999). The acute PCP pathway is not LRP- or β-catenin-dependent because DVL has multiple domains that can bind to different proteins. The combination of DVL and FZDs leads to the activation of different downstream signals (Jenny, 2010).

Six core genes, in addition to *Wnt*, *Fzd*, and *Dsh*, are involved in the PCP signaling pathway: strabismus (also known as Van Gogh), the cross-membrane co-receptor of the FZD protein, Prickle (the partner of Strabismus in the cytoplasm), and the small G-protein in the cytoplasm. RhoA and DSH can activate the Rac protein by binding their receptors, Rho-kinase (ROCK) and JNK, respectively.

**Fig. 3:**
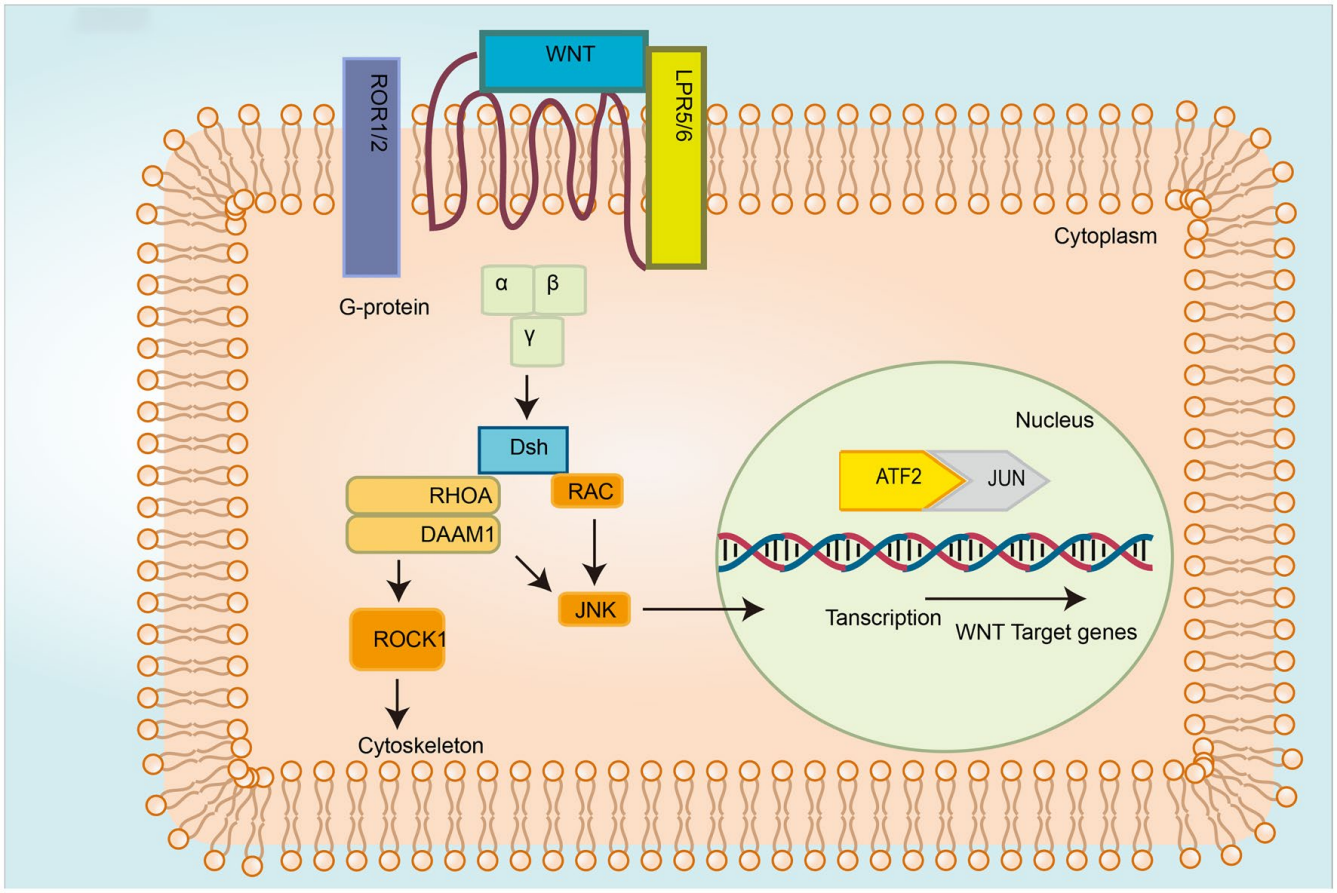
Overview of the non-canonical Wnt signaling pathways (Wnt/PCP). Upon interaction between the Wnt ligand and the receptor complex, FZD is recruited by DVL, and leads to the activation of disheveled associated activator morphogenesis-1 (Damm1) and the phosphorylation cascade. DVL activates RhoA and its downstream effector ROCK. DVL can also activate Rac1 and its downstream target JNK to initiate the transcription of target genes

The Wnt/PCP ligands, including Wnt5a, Wnt7, and Wnt11, bind to the FZD receptor. DVL-mediated induction of small GTPase (i.e., Rho and Rac) activates JNK and the transcription of downstream effectors involved in cytoskeletal rearrangement and epithelial cell polarization (Yang & Mlodzik, 2015). Rac and Rho can simulate kinases, such as Rho-kinase and JNK, leading to the activation of c-Jun N-terminal kinase and transcription factor 2 (Slusarski et al., 1997). The Wnt-FZD complex binds to Ror2 or RYK (Sergei, 2015) (Fig. 3).

#### The Wnt/Ca^2+^ Signaling Pathway

The second atypical Wnt signaling pathway is the Wnt/Ca^2+^ pathway, in which Ca^2+^ is released by the G-protein. The Wnt/Ca2^+^ pathway was originally found in nematode and zebrafish embryos (Ault et al., 1996; Slusarski et al., 1997). The Wnt/Ca^2+^ signaling pathway mediates cytoskeleton dynamics and cell adhesion by regulating intracellular Ca^2+^. The activation of the phospholipase C (PLC) leads to the release of intracellular Ca^2+^ and the subsequent activation of three Ca^2+^ sensitive components: protein kinase C (PKC) (Hu, 2020), calcineurin, and Ca^2+^/calmodulin-dependent kinase II (CaMKII) (Fuerer et al., 2008; Michael et al., 2000).

**Fig. 4:**
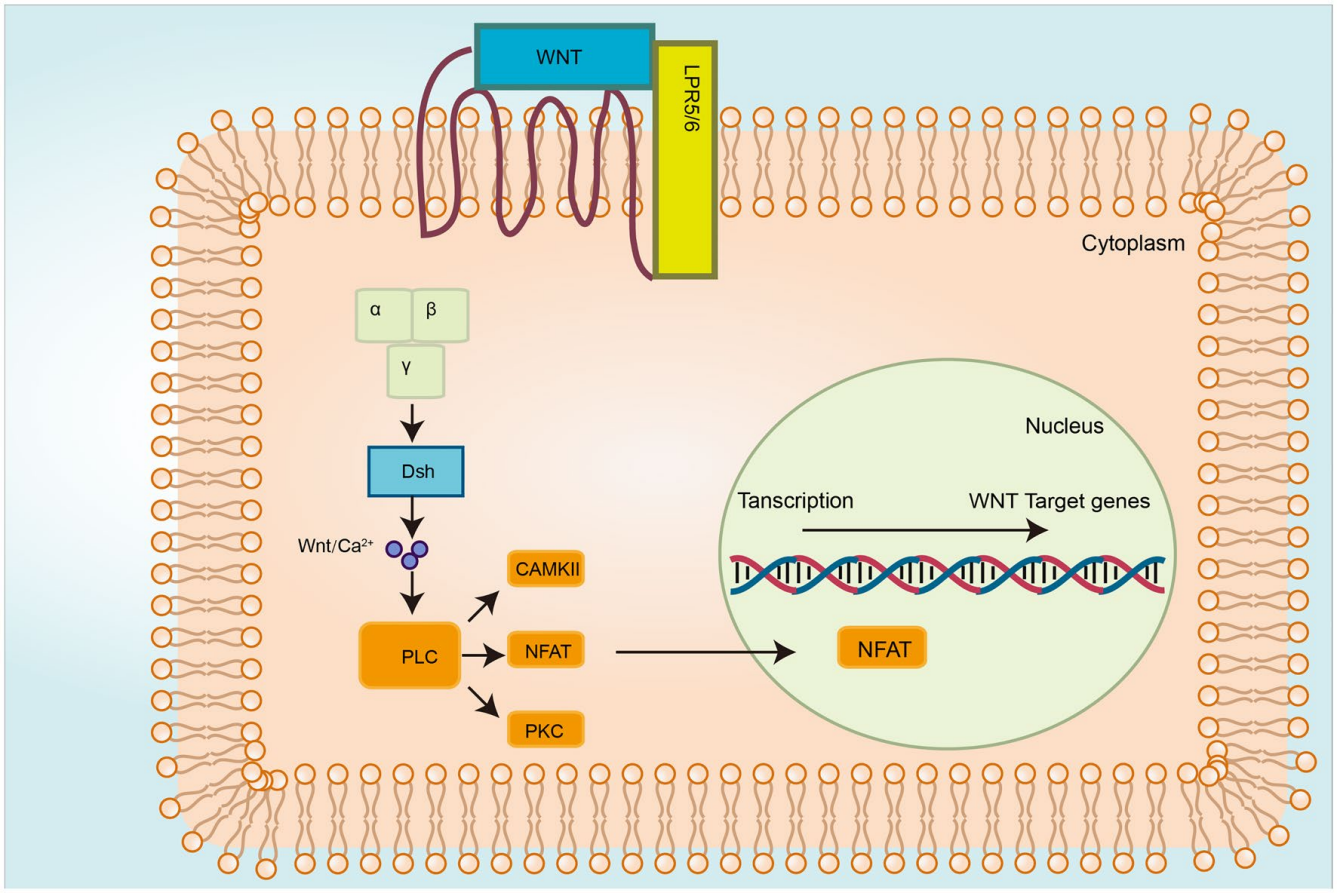
Overview of the non-canonical Wnt signaling pathways (Wnt/Ca^2+^). Activated FZD receptors recruit G-proteins, inducing the release of intracellular Ca^2+^. An elevation in Ca^2+^ level results in the activation of Ca^2+^-dependent signaling molecules, such as PKC, CAMKII and NFAT

The Wnt protein binds to FZD, recruits DVL, and activates phospholipase C, leading to the release of intracellular Ca^2+^, which in turn activates Ca^2+^ sensitive components PKC and CaMKII (Michael et al., 2000). The binding of Wnt to FZD receptors results in the activation of G-protein and the release of intracellular Ca^2+^. Through the Wnt/Ca^2+^ signaling pathway, the nuclear factor of activated T cells (NFAT) enters the nucleus, initiating the transcription of target genes (Fig. 4). The Wnt/Ca^2+^ signaling pathway also regulates the transcription of histone deacetylase 4 and mouse embryonic fibroblast (Niehrs & Christof, 2012; Zhang et al., 2015b).

## The Role of the Wnt Signaling Pathway in the Nervous System

The Wnt signaling pathway plays a vital role in maintaining the function of the nervous system. Emerging evidence suggests that Wnt signaling is involved in inflammation-induced brain damage and the corresponding repair process (Shruster et al., 2012; Maiese et al., 2012). The aberrant activation of the Wnt signaling is observed in CNS disorders caused by degeneration and inflammation of the mammals. The Wnt ligands and other Wnt signaling pathway-related components have also been found to be upregulated in the immune system of mammalian and immune-like cells (Halleskog, 2010; L'Episcopo et al., 2011b; Staal et al., 2008). The continuous expression of Wnt proteins maintains brain cell integrity (Mastroiacovo et al., 2008; Toledo et al., 2008).

The interaction between the immune system and the CNS is involved in neuron homeostasis, neuron repair, neurogenesis, and other physiological processes in response to injury (Martino et al., 2011). The activation of the Wnt signaling pathway is a crucial marker of neuronal inflammation and oxidative stress in the elderly with CNS diseases. Wnt signaling can be regulated by the interaction between macrophages and Glia cells (Halleskog, 2010; L'Episcopo et al., 2011a; Testa et al., 2011). In 2013, Marchetti et al. reported that Wnt signaling was involved in inflammatory responses in the CNS via interactions between macrophage/microglia and astrocytes (Marchetti & Pluchino, 2013). Other studies have shown that the activation of the Wnt signaling pathway in the nervous system may be responsible for the pathogenesis of certain CNS disorders, such as cerebral ischemia (Mastroiacovo et al., 2009), mental disorders, Parkinson’s disease (Toledo et al., 2008), Alzheimer’s disease (Adi et al., 2012; Halleskog, 2010), and epilepsy (Busceti et al., 2007). However, some reports have also indicated that constitutive activation of the Wnt pathway inhibits inflammation and promotes nerve formation (Halleskog, 2010; L'Episcopo et al., 2011b; Testa et al., 2011).

Taken together, these previous works indicate that the Wnt pathway plays a vital role in the physiological and pathological processes of the nervous system. WNT signaling pathway may be a potential mechanism for treating NP.

## The Wnt Signaling Pathway and NP

The main symptoms of acute and chronic NP are similar and include numbness and insensitivity in the extremities and even motor dysfunction in severe cases. NP is a common phenomenon in spinal cord injury, multiple sclerosis, diabetes, and other metabolic disorders. Cancer patients also experience NP caused by metastasis to peripheral nerves, tumors pressing on nerves or other organs, and chemotherapy. The Wnt signaling pathway plays a multifunctional role in nervous system development and the progression of NP (Hussain et al., 2019; Zhao et al., 2017; Zhong et al., 2018). This pathway is also closely related to central sensitization and the release of inflammatory factors, suggesting that the Wnt pathway may contribute to the pathogenesis of NP (Resham & Sharma, 2019).

Previous studies have shown that structural changes of neural circuits in the spinal dorsal horn (SDH) result in abnormal neuronal excitability through the release of neurotransmitters (Banafshe et al., 2014; Navarro et al., 2007; Woolf et al., 1992). Immune cells and Glia cells also participate in the development of NP. Currently available treatments for NP have limited efficacy. Therefore, it is of great significance to explore the mechanisms underlying the pathogenesis of NP and to develop novel therapeutic drugs (Ellis et al., 2013; Scholz & Woolf, 2007). The involvement of the Wnt pathway in the occurrence and maintenance of pain has been confirmed in many animal models. The activation of Wnt signaling in the SDH was observed in rodent pain models (Shi et al., 2012). The Wnt3a protein was upregulated in a mouse model of capsaicin-induced pain, while the blockage of this pathway alleviated pain (Zhang et al., 2018). Intrathecal injection of Wnt receptor inhibitors prevented NP or suppressed the pain already occurred (Itokazu et al., 2014). Some studies have also shown that the intrathecal injection of recombinant Wnt3a induces tactile allodynia. Such work has also found that activating the Wnt signaling pathway in the spinal cord (SC) is a common feature of NP.

The activation of the Wnt/β-catenin and Wnt/RYK signaling pathways enhances neuronal excitability by inducing the production of pro-inflammatory cytokines and the release of brain-derived neurotrophic factor (BDNF) and the activation of the Ca^2+^-dependent signaling pathways to enhance synaptic plasticity.

### The Chronic Constriction Injury (CCI) Model

The CCI animal model has been commonly used to mimic the symptoms of NP induced by tumor compression, heavy metal ion poisoning, and hypoxia. Animals experience hyperalgesia and spontaneous pain after surgery. The dorsal root ganglion (DRG) and SC damage is the key driver of NP development. Both typical and atypical Wnt signaling pathways are involved in regulating chronic sciatic nerve compression in rats. In the model of tumor cell implantation (TCI)-induced pain, the Wnt protein is upregulated in the DRG and SC, and the Wnt/FZD8/β-catenin signaling pathway is activated in the SC. The level of activated Wnt3a elevated in the superficial layer of the SDH after surgery (Zhang et al., 2013). Zhang et al. (2013) also observed rapid and constitutive expression of the Wnt protein family in SDH neurons and astrocytes in TCI- and CCI-induced pain models. The activation of the Wnt signaling induces NP, upregulates pro-inflammatory cytokines, such as interleukin (IL)-18 and tumor necrosis factor-α (TNF-α), and regulates the N-methyl-D-aspartate receptor 2B (NR2B) receptor through the β-catenin pathway in the spinal cord.

In the rat CCI model, the expression of transcription factor TCF4, a key component of the Wnt/β-catenin signaling pathway, is upregulated in the SC. In addition, Zhao et al. (2018) found significantly upregulated Wnt signaling molecules in the CCI model. This was accompanied by the accumulation of β-catenin in the nucleus and the increased production of IL-1, IL-8, and TNF-a, which leads to central sensitization and a decreased pain threshold. These findings confirm that the activation of the Wnt/β-catenin signaling pathway is involved in the pathogenesis of NP. In pain models, the Wnt signaling pathway is widely activated in DRG and SDH neurons. The expression of β-catenin, FZD4, and NR2B, and the phosphorylation level of CaMKII, PKC, cAMP-response element binding protein (CREB), and steroid receptor coactivator (Src) were significantly increased in the DRG after nerve injury. Moreover, intrathecal injection of the Wnt3a inhibitor IWP-2 significantly increased the mechanical pain threshold of CCI rats, and reduced the expression of β-catenin, FZD4, NR2B, p-CaMKII, PKC, CREB, and Src.

Recently, the regulation of the atypical Wnt signaling pathways through the RYK receptor has been reported (Yang et al., 2017). RYK contains a Wnt inhibitory factor-1-like extracellular structure domain, which enables the interaction with different Wnt ligands, including Wnt1 (Schmitt et al., 2006). Liu et al. (Liu et al., 2015) found that RYK and Wnt5b were upregulated in the SC and DRG of CCI rats. Intrathecal injection of the RYK antibody alleviated allodynia and hypersensitivity, indicating that the Wnt/RYK signaling pathway plays an important role in menstrual pain.

Substance P (SP) is a nociceptive transmitter in normal animals. Among the main afferents, it is mainly expressed by C fiber neurons (Lawson et al., 1997).

The CCI-induced over-excitation of the DRG neurons mainly manifests as the decrease in the action potential current threshold (APCT) and the increase in repeated discharge (Song, 2006). The RYK antibody has been shown to reverse the decline of APCT, reduce repetitive firing induced by intracellular depolarization current, and inhibit neuronal excitability. The RYK antibody blocks the RYK protein in the DRG and SC. It also significantly blocks C fibers, NR2B activation, and the activity of the Ca^2+^-dependent signaling proteins, including Src, CaMKII, PKC, CREB, and extracellular regulated protein kinases (Liu et al., 2015). Central sensitization, which is closely related to long-term potentiation (LTP), is an important mechanism underlying the occurrence and development of NP. It has been reported that the intrathecal injection of the RYK antibody blocks high-frequency stimulated C fibers between SDH neurons in CCI rats (Liu et al., 2015). It has been suggested that non-invasive A_ fibers begin to express SP and release SP in the spinal dorsal horn after peripheral inflammation or nerve injury. This is helpful for ectopic pain (pain caused by non-harmful stimuli), which is typical of neuropathic pain following peripheral nerve injury (Woolf, 2004; Zieglgänsberger et al., 2005).

It has recently been reported that 2 out of 20 A-fiber neurons expressed SP after CCI of the sciatic nerve, compared with 13 in the control group. The difference was not significant in the number of neurons investigated. The two A-fiber neurons expressing SP had higher conduction velocity (Lawson et al., 1997).

In a larger sample of unknown DRG neurons, a slight increase in medium-size SP immune response neurons was seen in neuropathic animals compared to controls. Few large DRG neurons expressed SP before and after CCI. In conclusion, if a phenotypic switch expressing SP exists in fiber neurons after CCI, the number of neurons affected is relatively small.

In summary, the Wnt/RYK pathway regulates the activation of NR2B, the intracellular level of Ca^2+^, and the expression of Ca^2+^-dependent signaling proteins. This pathway, therefore, controls the excitability of afferent A-fiber, C fibers and SDH neurons after nerve injury. Synaptic plasticity eventually leads to central sensitization in the SC and the development of NP.

NR2B, CaMKII, PKC, CREB, and Src are closely related to synaptic plasticity and the formation of central sensitization, which is the cause of secondary pain outside the initial injury site (Zhang et al., 2013). Wnt3a is involved in the pathogenesis of CCI-induced NP. The binding of Wnt3a to the FZD4 receptor on the postsynaptic membrane leads to nuclear translocation of intracellular β-catenin, the transcription of downstream target genes and eventually synaptic plasticity, central sensitization and a decrease in pain threshold. The mRNA and protein levels of TNF-α and IL-18 are significantly increased in the SC of CCI rats and the Wnt pathway is inhibited by intrathecal injection of IWR-1-endo (IWR-1) or WNT scavenger Fz-8/Fc (Zhang et al., 2013). Chromatin immunoprecipitation (ChIP) analysis showed that the β-catenin antibody interacts with the promoter sequence of TNF-α and IL-18 in the SC of CCI rats (Zhang et al., 2013). This indicates that Wnt/β-catenin signaling may participate in the development of NP by promoting the release of pro-inflammatory factors. Therefore, the Wnt/β-catenin and Wnt/RYK signaling pathways play an essential role in NP (Yu et al., 2018).

### The L5 Spinal Nerve Ligation (SNL) Model

The L6 transverse process is removed in the SNL model, and the L5 spinal nerve is tightly ligated without damaging the DRG or other nerves to induce mechanical hyperalgesia and thermal hyperalgesia.

The upregulation of Wnt3a and β-catenin in the SC has been reported in NP (Shi et al., 2012) and CCI models (Zhang et al., 2013), indicating that the activation of the Wnt/β-catenin signaling pathway in the SC is a common feature of NP.

Previous studies have shown that Wnt3a and β-catenin are upregulated in the SC of SNL mice (Tang, 2017). Intrathecal injection of the Wnt inhibitor XAV939 promoted β-catenin phosphorylation, selectively inhibited the Wnt/β-catenin pathway, and effectively relieved pain hypersensitivity. These findings indicate that the Wnt/β-catenin pathway is involved in the development of SNL-induced NP. Microglial BDNF has been shown to mediate the pathogenesis of NP (Scholz & Woolf, 2007; Siniscalco et al., 2011; Trang et al., 2011; Tsuda et al., 2005). The treatment of microglia with Wnt3a promoted the release of BDNF in vitro, and the upregulation of Wnt3a also elevated the expression of BDNF in the SDH in vivo. However, the intrathecal administration of XAV939 inhibited the activation of microglia, suggesting that Wnt3a promoted the activation of microglia, induced the secretion of BDNF, and eventually led to the occurrence of NP by activating the Wnt/β-catenin signaling pathway.

At 3 to 14 days after SNL, animals showed significantly upregulated mRNA and protein levels of RYK in SDH and increased protein expression of Wnt1 (RYK ligand) in astrocytes compared to the sham-operated group. Short hairpin RNA against RYK reduced inflammation and increased the threshold of mechanical pain in SNL-treated animals, indicating the implication of the Wnt1/RYK pathway in NP. The protein level of chemokine CCL2 in the SDH of a rat SNL model was significantly upregulated, while intrathecal injection of the RYK antibody reduced the production of CCL2 (Yang et al., 2017). These effects suggest that CCL2 may be the downstream target of the Wnt1/RYK signaling in the SC. In summary, these studies implied that the Wnt signaling pathway is closely related to the occurrence and development of NP.

### Human Immunodeficiency Virus (HIV)-Related NP

HIV-related NP is one of the most common neurological complications in HIV-1/AIDS patients (Aouizerat et al., 2010). It has been shown that the protein expression of Wnt5a, Wnt3a, Wnt4, Wnt9b, β-catenin, and axis inhibition protein 2 are significantly upregulated in the SDH of pain-positive HIV patients, but no significant changes are observed in pain-negative HIV patients (Shi et al., 2013b). Therefore, the activation of the Wnt pathway plays an important role in HIV-induced neuralgia. HIV-1 gp120 is a viral coat protein often used to simulate HIV-related NP in mice. Wnt3a is significantly upregulated in the microglia of HIV-1 gp120-treated mice, and the functional status of microglia is directly related to HIV-induced neuropathic pain (HNP) (Coull et al., 2005; Wang et al., 2017b). In the SDH of mice with HIV-1 gp120-induced neuralgia, the expressions of Wnt5a, IL-1β, IL-6, and TNF-α were upregulated. At the same time, the intrathecal injection of Wnt5a antagonist Box5 significantly reduced the levels of IL-1β, IL-6, and TNF-α compared with the control group (Shi et al., 2013b). Research also found that HIV-1 gpl20 induced the expression of pro-inflammatory cytokines and induced HNP in mouse SDH through the Wnt5a/CaMKII and Wnt5a/JNK signaling pathways.

NMDA is a key mediator of Wnt5a (Farias et al., 2009), which plays a critical role in the differentiation and plasticity of exciting synapses (Li et al., 2013; Varela-Nallar et al., 2010). Excessive activation of the SDH neurons under pathological pain conditions induces the expression of Wnt5a, thereby promoting the release of inflammatory cytokines (i.e., IL-1β, IL-6, and TNF-α) and the synaptic plasticity of SDH neurons, and eventually induces HNP.

In mice with gp120-induced pain, the protein expression of Wnt5a in SDH neurons was upregulated and the phosphorylation levels of CaMKII and JNK protein were significantly increased compared to the controls (Li et al., 2013). Intrathecal injection of Wnt5a antagonist Box5, however, suppressed gp120-induced phosphorylation of CaMKII and JNK (Li et al., 2013), indicating that gp120 promoted the phosphorylation of CaMKII and JNK by upregulating Wnt5a. The KN-93 and SP600125 inhibitors were recently used to inhibit the expressions of CaMKII and JNK in gp120-treated SDH. The results showed that KN-93 inhibited gp120-induced upregulation of IL-6 and IL-1β, while SP600125 inhibited the increase in IL-6 and TNF-α expression (Li et al., 2013). These data suggest that CaMKII promotes the release of IL-6 and IL-1β, and JNK induces the production of IL-6 and TNF-α. To sum up, gp120 induces the activation of the Wnt5a/CaMKII and Wnt5a/JNK pathways, leading to the release of inflammatory cytokines and the development of NP.

### Cancer Pain (Tumor-Associated Pain)

The mouse model of bone cancer pain induced by TCI is commonly used to investigate mechanical and thermal hyperalgesia. The protein expression of Wnt3a, Wnt receptor FZD8, and β-catenin are significantly increased at the surgical site of the SC (Zhang et al., 2013).

Pain is one of the most common symptoms of cancer patients and seriously affects their quality of life. The incidence of pain in newly diagnosed cancer patients is 25%, while the incidence in advanced cancer patients is about 60–80%, of which 1/3 of the patients have severe pain (Ministry of Health of the PRC, 2011). The WHO has developed a “three-step” treatment plan for cancer pain, but the long-term use of analgesics is associated with decreased analgesic effects. Morphine was the first alkaloid isolated from opium by a German chemist in 1806. Morphine hydrochloride is a commonly used analgesic for severe trauma, surgery, burns, and moderate to severe pain caused by advanced cancer. However, morphine tolerance and hyperalgesia may develop after long-term use, and the side effects of morphine are challenging to manage. When morphine is administered, the level of Wnt3a and Wnt5a in the SC and DRG tissues and the expression of receptors FZD1 and FZD8 are significantly upregulated, implicating the involvement of the Wnt signaling pathway in morphine tolerance and hyperalgesia (Li, 2016).

A scorpion peptide is reported to have both analgesic and antitumor activity in animal models and may be an alternative treatment for breast cancer (Shao et al., 2014; Wang et al., 1994).This study aimed to investigate the effects of Buthus martensii Karsch antitumor-analgesic peptide (BmK AGAP) on stem cell differentiation and epithelial–mesenchymal transition (EMT) of breast cancer cells. Here we demonstrate that RBMK AGAP downregulates PTX3 through NF-κB and Wnt/β-catenin signaling pathways in vivo and in vitro, thereby inhibiting dryness, EMT, migration, and invasion of breast cancer cells. The xenograft model confirmed that RBMK AGAP inhibited tumor growth, stem-like features, and EMT. It has been reported that BMK AGAP has analgesic and antitumor effects (Li et al., 2016; Liu et al., 2003; Ruan et al., 2018). Many animal studies have confirmed the analgesic activity of BMK AGAP. Therefore, RBMK AGAP is a potential treatment for breast cancer and associated pain (Kampo et al., 2007).

Current studies have found that the infiltrated dendritic cells in tumor microenvironment can produce hormones. They secrete paracrine factors such as tumor necrosis factor (TNF), WNT10A, platelet-derived growth factor alpha (PDGFA), and neuregulin 1 (NRG1) to sensitize sensory neurons, thereby promoting neuropathic pain associated with various cancers. The interaction between paracrine factors and their receptors can cause the activation of downstream transcription factors such as B-Cell lymphoma 3-encoded protein (BCL3), E2F transcription Factor 1 (E2F1) and SMAD family member 5 (Smad5), and RE1 silencing transcription factor (REST) can upregulate the expression of pain-related genes such as chromodomain helicase DNA binding protein 5 (CHD5) and pain including arrestin beta 2 (ARRB2). TNF, WNT10A, and Platelet-derived growth factor alpha (PDGFA) are widely expressed in various tumors, but their expression is not normally distributed in patients. These data indicate the presence of infiltrating dendritic cells in the tumor. The microenvironment promotes neuropathic pain by sensitizing nociceptive sensory neurons with paracrine factors. Blocking paracrine signaling may alleviate cancer pain (Wang et al., 2020).

### Diabetic NP (DNP)

Diabetes is a common metabolic disease accompanied by mild inflammation (Ganesan, 2019), and NP is one of the severe complications of this disease (Banafshe et al., 2014). Approximately one-third of diabetic patients suffer from diabetic neuropathy, and the main manifestations include a burning sensation on the skin of the lower extremities, spontaneous pain, and hyperalgesia. Streptozotocin (STZ) is a chemical inducer of diabetes that causes toxicities, such as oxidation and hydroxylation. The STZ model is widely used to investigate experimental DNP. Compared with a control group, rats with STZ-induced diabetes exhibited a significantly increased mechanical pain and reduced thermal pain threshold (Zhong et al., 2018). The protein expression of Wnt10a and β-catenin in the SC and the level of TNF-α and IL-1β were significantly increased in the STZ model, indicating that Wnt10a may induce the production of TNF-α and IL-1β by activating the Wnt/β-catenin signaling pathway during the pathogenesis of DNP.

Diabetic peripheral neuropathy (DPN) is a common complication of diabetes that affects more than half of diabetics throughout disease progression, leading to neuropathic pain and an increased risk of ulcers and amputations (Pop-Busui et al., 2017). Demyelination is the most typical feature of DPN pathologic development and is closely associated with neurological dysfunction and reduced nerve regeneration (Zenker et al., 2013). Therefore, myelin regeneration is the key to promoting peripheral nerve function recovery and alleviating DPN symptoms.

β-catenin and PI3K/AKT pathways play important roles in DPN. However, high glucose can reduce the expression of β-catenin (Wang et al., 2017a).Notoginsenoside R1 (NGR1) is a major bioactive ingredient of Radix notoginseng. Mir-503 is a miRNA sensitive to hyperglycemia and can be highly expressed in diabetic patients (Caporali et al., 2011). In addition, Mir-503 is considered a factor regulating nerve injury (Saba et al., 2019), suggesting that it is a potential therapeutic target for DPN.

Research found that Notoginsenoside R1 (NGR1) can activate PI3K/AKT and B-catenin signaling pathways by downregulating Mir-503 under high glucose conditions (Li et al., 2018b, 2019).

This finding suggests that NGR1 may confer anti-DPN properties by downregulating Mir-503 and then modulating the two signals.

Tang-luo-ning (TLN) is a traditional Chinese medicine prescription for treating diabetic peripheral neuropathy (DPN). Relevant studies studied the effects of TLN on microRNA (MiRNA) expression in diabetic rats, and predicted the target genes.

Further pathway and genetic analysis identified DVL1 from the Wnt signaling pathway and neurotrophin-3 (NTF3) from the neurotrophin signaling pathway. Previous studies have shown that Wnt signaling pathway may mediate oxidized low-density lipoprotein (ox-LDL)-induced endothelial cell injury through oxidative stress. That inhibition of oxidative stress and inhibition of Wnt signaling pathway can ameliorate endothelial cell injury (Ma et al., 2017). The research suggests that TLN may affect the expression of DVL1, and then affect Wnt signal transduction, which may alleviate oxidative stress and improve DPN symptoms.

Jinmaitong (JMT), is a commonly used compound medicine. It consists of 12 kinds of Traditional Chinese medicine, including 10 types of Chinese medicinal materials and two types of animal medicinal materials, containing a variety of components with unique chemical structures and a variety of biological characteristics. Previous studies have verified the alleviating effect of the components on diabetic peripheral neuropathy (DPN). Among them, quercetin, cinnamaldehyde, and hirudin come from three main herbs (Semen Cuscutae, Ramulus Cinnamoml, and Hirudo, respectively) respectively, and their neuroprotective effects have been verified. As a result, all of these compounds have protective effects on rat dorsal root ganglion (DRG) neurons against in vitro damage induced by high glucose (Li et al., 2020; Shi et al., 2013a).

In the present study, the neuroprotective effect of JMT was confirmed on DPN rats. Then during DPN progression and at 8, 12, and 16 weeks of JMT treatment, the expression of β-catenin, glycogen synthase kinase 3β (GSK-3 β), and WNT inhibitory factor-1 (WIF-1) involved in WNT /β-catenin signaling pathway and Myelin protein zero (MPZ) in rat sciatic nerve was determined.

The results showed that the expression of β-catenin mRNA and protein was significantly inhibited after sciatic nerve injury in rats. After 8, 12, 16 weeks, the mRNA and protein levels of β-catenin were increased considerably, and the expression of P-β-catenin decreased. These results are consistent with our previous in vitro studies, which found that JMT promotes myelin repair by stimulating Schwann cell proliferation in high glucose medium and enhances MPZ secretion by upregulation of β-catenin (Sun et al., 2017).

Therefore, our in vivo and in vitro studies showed β-catenin upregulation and p-β-catenin downregulation after JMT treatment, suggesting that the WNT /β-catenin signaling pathway activation is at least partially involved in the neuroprotective effect of JMT on peripheral neuropathy.

This study was to observe the protective effect of adding Traditional Chinese medicine on diabetic peripheral neuropathy, the protective effect of different courses of treatment, and different doses of JMT on diabetic peripheral neuropathy. Meanwhile, it was found for the first time that the inhibited Wnt/β-catenin pathway was activated by JMT in a time-dependent manner in the sciatic nerve of diabetic nephropathy rats, which may lead to upregulation of MPZ level and regeneration of peripheral nerves (Wang et al., 2019).

### Multiple Sclerosis (MS)-Related NP

MS is an autoimmune disease characterized by inflammatory demyelination of the white matter of the CNS. More than 80% of MS patients will develop NP (Ruan et al., 2018). The pathological signs of MS include chronic inflammation, blood–brain barrier destruction, and demyelination. Chronic inflammation is the main cause of myelin sheath destruction in the brain and SC axons, leading to progressive loss of nerve function and neuronal apoptosis. The inflammatory process is initiated by microglia in the acute phase of MS and mediated by CD4^+^T helper (Th) cells Th1 and Th17 in the chronic phase. Th17 cells are the main immune participants involved in the pathogenesis of MS by mediating the production of pro-inflammatory cytokines (i.e., IL-17, IL-6, IL-21, IL22, IL-23, and TNF-α) (Jadidi-Niaragh & Mirshafiey, 2011). In a mouse MS model, the absence of LRP5, LRP6, or β-catenin in dendritic cells leads to the enhanced release of pro-inflammatory cytokines and the increased polarization of Th1 and Th17 cells, which aggravates the disease. On the contrary, the activation of the β-catenin signaling pathway in dendritic cells, through TLR2 signaling, inhibits the release of pro-inflammatory cytokines (Manoharan et al., 2014). Therefore, the activation of the Wnt/β-catenin signaling pathway promotes immune tolerance of dendritic cells, inhibits T-cell differentiation, and reduces autoimmune neuroinflammation in MS (Manoharan et al., 2014). A previous study also suggested that the canonical Wnt signaling pathway inhibits remyelination, which involves the recruitment of oligodendrocyte precursor cells to the lesion sites, cell proliferation, cell differentiation to mature oligodendrocytes, and myelin restoration (Xie et al., 2014).

Experimental allergic encephalomyelitis (EAE) mice have been widely used to investigate MS-related neurological complications, including central nerve demyelination, neuroinflammation, movement disorders and sexual pain. Immunohistochemistry analysis showed that Wnt3a, β-catenin, Wnt5a, and its receptor Ror2 were significantly upregulated in the SDH of EAE mice. Intrathecal injection of Wnt5a antagonist Box5 and β-catenin inhibitor indomethacin effectively alleviated pain in EAE mice (Yuan et al., 2012), suggesting that the activation of the typical or atypical Wnt signaling pathways may promote the occurrence of EAE-related pathological pain. Previous data have also revealed that Wnt5a is a key regulator of the expression of pro-inflammatory cytokines, such as IL-1β and TNF-α, which are important mediators of neuroinflammation in pain (Dominik, 2011; Kiguchi et al., 2012).. Therefore, the Wnt5a signaling may be involved in the pathogenesis of pathological pain in EAE mice by upregulating the expression of pro-inflammatory cytokines.

### Myasthenia Gravis (MG)

MG is an autoimmune disease involving impaired neuromuscular transmission and weakness of skeletal muscles (Dominik, 2011). The Wnt/β-catenin signaling pathway is implicated in the formation of neuromuscular junctions (Messéant et al., 2017). Skeletal muscle contraction is controlled by the neurotransmitter acetylcholine (ACh), which is released from the motor nerve endings. The density of the ACh receptors (AChR) on the muscle endplates must be high (> 10,000 AChR/μm^2^) to ensure that muscle membrane potential depolarization can be trigged. Receptor-associated protein at the synapse (Rapsyn) is a protein that promotes the aggregation of acetylcholine receptors (Chen et al., 2016). The activation of the Wnt/β-catenin signaling pathway inhibits the accumulation of AChR at the neuromuscular junction by blocking the expression of Rapson. A recent study showed that the use of R-spondin2, a Wnt/β-catenin signaling pathway agonist, promoted the accumulation of AChR and the formation of neuromuscular junctions through its receptor LGR5 (Li et al., 2018a).

### The Relationship Between the Wnt/β-Catenin Signaling Pathway and Endometriosis (EMT)

EMT is a pathological condition characterized by uncontrolled proliferation and distant metastasis of blood vessels and other circulatory system components to surrounding tissues (Lang, 2004). Estrogen imbalance induced by both internal and external factors is the main cause of EMT (Chen, 2018; Zhang, 2007).

The Wnt/β-catenin signaling pathway is involved in ectopic endometrial adhesion, invasion, angiogenesis, and inflammation during the progression of EMT (Liu et al., 2010; Mattos et al., 2016; Zhang et al., 2015a). It has been shown that the inhibition of GSK-3β significantly promotes the occurrence and development of EMT (Zhang et al., 2015a). When β-catenin enters the nucleus, instead of combining with TLE/Groucho, it combines with lymphoid enhancer factor (LEF) and T-cell factor (TCF) complexes, recruits CBP/P300, BRG1, BCL9, Pygo, and other co-activators, and then upregulates the expression of target genes. Liang et al. (Liang et al., 2012) found that the intervention of the Wnt/β-catenin signaling pathway in vivo changed the invasion and metastasis characteristics of the endometrium, its ability to adhere to blood vessels and other tissues, and caused an alteration of the endometrium from the normal to an ectopic position. A recent study reported that aberrant expression of MSI-1 was involved in the activation of the Wnt/β-catenin signaling pathway in EMT (Sanchez et al., 2014). In addition, the transcription level of Wnt4 and Wnt5a in the ectopic endometrium of EMT patients are significantly higher. In comparison, the level of Wnt1 is significantly lower than that in healthy women, indicating that altered expression of Wnt molecules is related to the onset of EMT (Zhou, 2014). The upregulation of β-catenin in the nucleus has been observed in around 30% of estrogen-related tumors. Therefore, the Wnt signaling pathway may be used as a therapeutic target for EMT.

## Conclusion

As an important regulatory pathway of embryonic development and adult tissue homeostasis, Wnt signaling pathway is closely related to a variety of diseases and is an important target of drug therapy. A growing body of evidence supports the role of Wnt signaling in regulating NP and has been validated in many animal models. Studies have shown that WNT may be involved in the pathogenesis of NP by regulating central sensitization and inflammatory factor release. Application of WNT signaling pathway-related blockers can effectively alleviate NP.

Further investigations on the Wnt signaling pathway may contribute to developing novel therapeutic options for NP patients, thereby improving their quality of life.